# The Perceived Benefits of an Artificial Intelligence–Embedded Mobile App Implementing Evidence-Based Guidelines for the Self-Management of Chronic Neck and Back Pain: Observational Study

**DOI:** 10.2196/mhealth.8127

**Published:** 2018-11-26

**Authors:** Wai Leung Ambrose Lo, Di Lei, Le Li, Dong Feng Huang, Kin-Fai Tong

**Affiliations:** 1 Guangdong Engineering and Technology Research Center for Rehabilitation Medicine and Translation Department of Rehabilitation Medicine The First Affiliated Hospital, Sun Yat-sen University Guangzhou China; 2 Department of Electronic and Electrical Engineering University College London London United Kingdom

**Keywords:** low back pain, neck pain, mobile app, exercise therapy, mHealth

## Abstract

**Background:**

Chronic musculoskeletal neck and back pain are disabling conditions among adults. Use of technology has been suggested as an alternative way to increase adherence to exercise therapy, which may improve clinical outcomes.

**Objective:**

The aim was to investigate the self-perceived benefits of an artificial intelligence (AI)–embedded mobile app to self-manage chronic neck and back pain.

**Methods:**

A total of 161 participants responded to the invitation. The evaluation questionnaire included 14 questions that were intended to explore if using the AI rehabilitation system may (1) increase time spent on therapeutic exercise, (2) affect pain level (assessed by the 0-10 Numerical Pain Rating Scale), and (3) reduce the need for other interventions.

**Results:**

An increase in time spent on therapeutic exercise per day was observed. The median Numerical Pain Rating Scale scores were 6 (interquartile range [IQR] 5-8) before and 4 (IQR 3-6) after using the AI-embedded mobile app (95% CI 1.18-1.81). A 3-point reduction was reported by the participants who used the AI-embedded mobile app for more than 6 months. Reduction in the usage of other interventions while using the AI-embedded mobile app was also reported.

**Conclusions:**

This study demonstrated the positive self-perceived beneficiary effect of using the AI-embedded mobile app to provide a personalized therapeutic exercise program. The positive results suggest that it at least warrants further study to investigate the physiological effect of the AI-embedded mobile app and how it compares with routine clinical care.

## Introduction

Chronic musculoskeletal neck and back pain are prevalent and disabling conditions among adults [1]. The report on global economic burden of disease published in 2010 indicated low back pain and neck pain ranked sixth and 21st, respectively, out of 291 diseases and injuries [2] for medical cost. The studies published in 2016 indicated that combined low back and neck pain ranked fourth out of 315 diseases and injuries [3] for causes of years lived with disability. Thus, there is an urgent need to identify an effective means to promote self-management of these conditions.

Recent advancement in information and communication technology (ICT) places strong emphasis on supporting self-management programs for chronic pain through mobile software apps [4,5]. Mobile apps have been identified as a way to reduce the financial burden of chronic neck and back pain [6]. Use of ICT as part of rehabilitation programs has been suggested to increase adherence to self-management programs [7]. However, published studies have indicated that approximately 65% of the neck and back pain-related mobile apps have no evidence of the involvement of health care professionals [8,9] in their development.

To date, only one study was found that investigated the benefits of a professionally designed mobile app that had been trialed. This study tested the effect of using a mobile app on and its benefit in self-management of low back pain [10]. The app was designed with a group of pain professionals and utilized a cognitive behavior approach based on recommendations from the American Pain Society [11]. A total of 597 participants with chronic low back pain who had not received any intervention for the condition were recruited. They were randomly assigned to intervention, routine care, and control group. Outcome measures included level of pain and pain frequency. Results indicated greater improvement at 4-month follow-up in both parameters compared to the control group. This study demonstrated that a mobile app may be an effective tool in self-management of low back pain. Although this study provided positive results for the use of mobile app, it excluded the majority of people with chronic back pain that had some sort of prior intervention. In addition, the exercise program appeared to be generic exercises rather than ones specific to the users’ symptoms.

Other studies are also trialing different mobile apps that focus on relaxation therapy [12], with a combination of mobile app and clinician offline support [6]. Results of these studies are yet to be published. Currently, there are a large number of health-related mobile apps on the market, but the majority of the neck and back pain apps have focused on pain management education. Other mobile apps claim to offer exercises and education for chronic neck and back pain but provide limited evidence that a health care professional was involved in their development [13].

Our institute has taken part in developing an artificial intelligence (AI) system embedded into a mobile app called “Well Health.” The AI-embedded mobile app was developed in response to the “World Report on Disability” [14], which suggested that electronic health (eHealth) or telerehabilitation techniques are effective means to enable people to receive appropriate intervention. However, it also stated that telerehabilitation should be tested to assess its feasibility within the local culture. As documented in a survey of the Chinese population with chronic low back pain (N=113) in 2016, it was found that self-management behaviors were poor [15]. The survey identified that the contributing factors to poor self-management behavior included a lack of disease knowledge, a lack of understanding of the benefits of exercise, and a lack of communication with health professionals. The AI-embedded mobile app was designed to address some of these factors. It allows users to input their symptoms and generates an exercise program accordingly. The exercise program is delivered in the form of a video that plays in real time.

The aim of this study was to evaluate the feasibility of the AI-embedded mobile app Well Health to assist people with chronic neck and back pain to self-manage their condition, and the perceived benefits in symptom improvement by its users. This is the first study that evaluates an AI-embedded mobile app for neck and back pain rehabilitation among the Chinese population.

## Methods

### Ethical Consideration

The study was approved by the Medical Ethical Committee for Clinical Research and Animal Trials of the First Affiliated Hospital of Sun Yat-sen University (reg # [2016] 185). Informed consent was obtained electronically. Once the users clicked on the survey invitation icon, they were directed to an informed consent form. Informed consent was given by selecting “Agree to take part” and “Submit.” Each participant was assigned a number to maintain anonymity.

### Recruitment

This study was a retrospective evaluation study of an AI-embedded mobile app that was available to the general public. All existing users of the mobile app were invited to take part via the in-app “Information” section. The invitation also appeared on the home page where it would be displayed among five rolling pictures of different newsfeeds when the app was loaded each time. Interested users could click on the link which directed them to the evaluation questionnaire. All the participants in this study completed the questionnaire one time, with retrospective recall of preintervention pain level.

### Sample Population

The inclusion criteria for the study were adults (1) aged between 18 and 65 years, (2) who had experienced neck and low back pain within the past 3 months, and (3) had access to a mobile phone that could play video on the internet. This study excluded patients who were medically unstable and reported having red flags for cervical and lumbar spine pathology. A list of red flag symptoms related to the cervical and lumber spine were displayed when the app was opened the first time and before the start of the questionnaire. The user would not be able to continue with the survey if more than two red flag symptoms were selected. These inclusion criteria were set to include a wide population because the app was designed to be accessed by members of the general public with experience with chronic neck and back pain.

### Evaluation Questionnaire

The questionnaire included 14 questions that were intended to evaluate if using the AI-embedded mobile app may (1) increase adherence to therapeutic exercises, (2) affect pain level, and (3) reduce the need for other interventions. The World Health Organization defined the term “adherence” as “the extent to which patient behavior taking medication, following a diet, and/or executing lifestyle changes, corresponds with recommendations from a health care provider” in 2003 [16].

**The evaluation questions.**

1. Have you experienced neck and low back pain within the past 3 months? *Responses: (1) yes; (2) no*

2. Are you age between: *Responses: (1) 18-25; (2) 26-30; (3) 31-40; (4) 41-50; (5) 51-60; (6) 60-65?*

3. What is your gender? *Responses: (1) male; (2) female*

4. How long have you used the AI-embedded mobile app? Responses: *(1) 1 day; (2) 1 week; (3) 1 month; (4) 3 months; (5) 6 months or over*

5. On a scale of 0-10 (0 being no pain, 10 being in extreme pain), how would you rate your pain level before using the rehabilitation program on the AI-embedded mobile app? *Responses: 0 to 10 Likert scale*

6. On a scale of 0-10, how would you rate your pain level after using the AI-embedded mobile app for rehabilitation program? *Responses: 0 to 10 Likert scale*

7. Prior to using the app, have you participated in any rehabilitation exercise program? *Responses: (1) yes; (2) no*

If “Yes,” how much time did you spend on rehabilitation exercise a day on average? *Response: (1) 5 minutes or below; (2) 6 to 10 minutes; (3) between 10 and 30 minutes; (4) between 30 and 60 minutes; (5) 60 minutes or above*

8. How much time in a day have you followed the AI-embedded mobile app to perform the recommended exercise? *Responses: Amount of time entered by the user (value between 0 to 180 minutes)*

9. On a scale of 0-100 (0 being no improvement, 100 being completely resolved), how would you rate the overall improvement of your symptoms? *Responses: 0 to 10 Likert scale*

10. Have you received any of the following interventions prior to using the AI-embedded mobile app? *Responses: (1) acupuncture; (2) soft tissue therapy; (3) topical cream, (4) medication; (5) electrotherapy*

11. Have you received any of the following interventions prior to using the AI-embedded mobile app? *Responses: (1) acupuncture; (2) soft tissue therapy; (3) topical cream, (4) medication; (5) electrotherapy*

12. While using the AI-embedded mobile app, have you continued with any of the interventions mentioned? *Responses: (1) acupuncture; (2) soft tissue therapy; (3) topical cream, (4) medication; (5) electrotherapy; (6) no, I did not use any other intervention while using the AI-embedded mobile app*

13. Have you read the education material within the app? *Responses: (1) yes; (2) no.*

If “yes,” how much time in a day have you spent on reading the educational material? *Responses: amount of time entered by the user (value between 0 and 180 minutes)*

14. Would those materials encourage you to adhere to the therapeutic exercise? *Responses: (1) yes; (2) no*

Based on this original definition, Donkin et al [17] provided a further definition of “the degree to which the user followed the program as it was designed” to incorporate e-therapies in 2011. A systematic review on the chronic low back pain population also found self-report diaries to be the most common measure of adherence [18]. A published study indicated a self-reported exercise log has acceptable agreement with objectively assessed exercise adherence for both exercise frequency and exercise duration [19]. Therefore, the operating definition of adherence in this study was the amount of “self-reported time spent on therapeutic exercises in a day” because the AI-embedded mobile app was designed to encourage daily therapeutic exercise.

The evaluation questions are presented in Textbox 1. The first two questions were used to assess if the responses fit the inclusion criteria.

In addition to clinical information, participants were asked to fill in the modified System Usability Scale (SUS). The scale had 10 questions that were validated to assess the generic usability of a product or a service [20]. The questions are as follows:

1. I think that I would like to use this product frequently.
2. I found the product unnecessarily complex.
3. I thought the product was easy to use.
4. I think that I would need the support of a technical person to able to use this product.
5. I found the various functions in this product were well integrated.
6. I thought there was too much inconsistency in this product.
7. I would imagine that most people would learn to use this product very quickly.
8. I found the system very awkward to use.
9. I felt very confident using the product.
10. I needed to learn a lot of things before I could get going with the product.

The SUS showed the domains as five scales numbered from 1 (strongly disagree) to 5 (strongly agree). Positively worded domains were equal to score–1, negatively worded domains were equal to 5–score, so the total SUS was calculated by summing the 10 domains and multiplying by 2.5.

### Intervention: Well Health Mobile App

#### Credential and Affiliations of the Developers

The affiliations of the developers and the expert panel that were involved in developing the AI-embedded mobile app were clearly displayed within the “About us” section within the mobile app. The embedded AI system, including the assessment method, the exercise programs, and exercise instructions, were developed with an expert panel consisting of physiotherapists from Australia (Maxvale Physiotherapy Practice), the United Kingdom (University College London), and China (Sun Yat-sen University), including a clinical scientist and a rehabilitation doctor from China (Sun Yat-sen University). Technical support in developing and maintaining the app and AI coding was done in collaboration with the information technology company “Well Health” (Ying Kang Wei Er Internet Service Co, Ltd, Guangzhou, China).

#### Development Process

The AI-embedded mobile app underwent 10 months of development. Members of the expert panel met in China for 3 weeks for residential development. During the residential period, the clinical content of the AI-embedded mobile app was determined. This included the development of the AI algorithm (initial training sample preparation, neuron processing methods), production of the exercise videos and their associated descriptions, and the education material. This was followed by 3 months of AI model validation. During this phase, experts went through computer-simulated data to ensure the AI-generated exercise programs were appropriate for the presented symptoms. The development of the mobile app interface took a further 6 months.

System usability and interface development of the app were conducted in the form of a semi-structured telephone interview (n=72) and focus group (n=8) prior to this study. The interview and focus groups were designed to gather information on (1) what users like to use the app for (eg, just browse for information or look for rehabilitation exercises for their back pain); (2) what additional functions they would like to have in the app; (3) how do they like the exercise content to be delivered (eg, exercises to be described by text or displayed as picture accompanied by text or in video with verbal guidance; (4) any in-app functions that users felt were missing; and (5) users’ feelings on the app interface. No update was made to the content of the AI-embedded mobile app during the data collection phase.

The AI-embedded mobile app is available free of charge online to the general public in the US, UK, and China through the Apple app store and MI app store. The app is searchable using the keyword “Well Health.”

#### Access

Access to the mobile app was completely free. The AI-embedded mobile app could be downloaded from any well-known app store. Participants were required to register an account using their mobile phone number or social media account. Technical support was provided by Well Health in registering for a user account, if needed.

### Intervention Components

#### Physical Component

Well Health is a multiple-visit mobile app that provides adults with neck and back pain a tailored exercise rehabilitation program based on their presenting symptoms.

**Figure 1:**
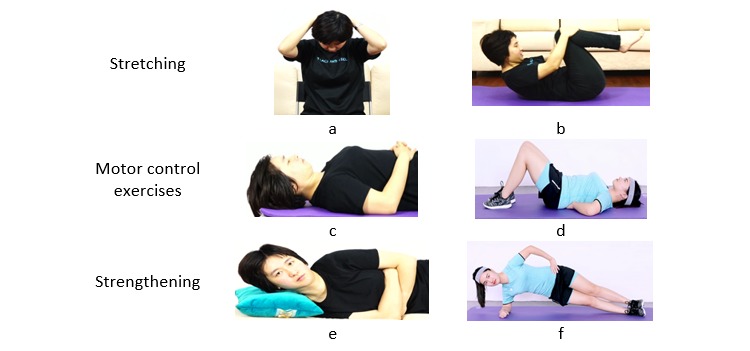
Screenshots of stretching, motor control, and strengthening exercises: (a) assisted neck flexion stretch, (b) lumbar flexion stretch, (c) deep neck flexors exercise, (d) transverse abdominis activation exercise, (e) cervical side flexion exercise, and (f) side-lying trunk exercise.

The AI-embedded mobile app follows the recommendations of the National Institute of Clinical Excellence (NICE) [21] in providing a combination of physical and self-management advice as well as the pathological causes of low back pain. It allows users to enter their symptoms using a self-report questionnaire. The questionnaire consists of the questions which physiotherapists routinely use to conduct a subjective assessment. The questionnaire asks for information on present condition, history of present condition, past investigations, 24-hour pattern, drug history, and social history. Once the subjective assessment is completed, the AI-embedded mobile app provides an exercise program based on the information provided. Exercise interventions provided by the AI-embedded mobile app were NICE guideline-recommended biomechanical exercises including stretching, (eg, lumbar flexion and neck flexion); motor control exercises, including Pilates exercises since their principles overlap with principles of motor control interventions [22] (eg, deep neck flexors exercise, transverse abdominis activation exercise); and strengthening exercises (eg, cervical side flexion exercise, side-lying trunk exercise). Figure 1 shows screenshots of these exercises. The suggested exercise duration was between 20 to 30 minutes a day.

Each exercise video was accompanied by detailed descriptions of the exercises and annotated diagrams were displayed to visually demonstrate the targeted muscles (see Multimedia Appendix 1). Users were expected to follow the video clip from start to finish once they started the set of training. The video clip could not be scrolled through, but could be paused/stopped. Points were awarded when the full length of the video was played.

**Figure 2:**
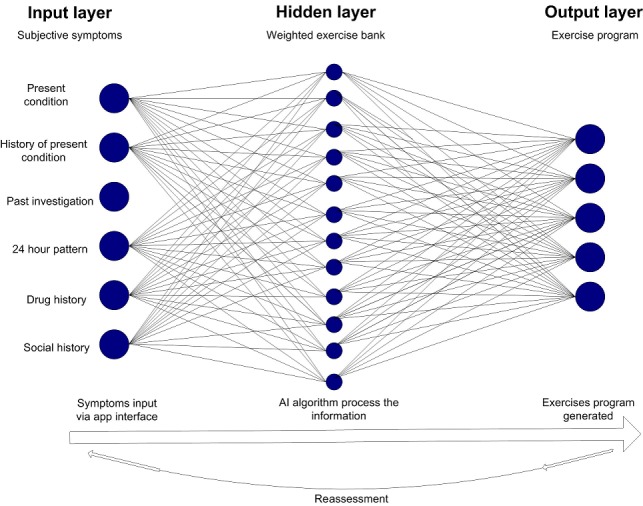
The architecture of the multilayered perceptron artificial neural network used in this study.

The AI-embedded mobile app determined the exercise program based on the subjective information. The AI algorithm used in this study was based on the multilayered perceptron artificial neural network (MLP-ANN). It is the most commonly used AI algorithm in the medical field [23] and is capable of learning from historical examples, analyzing nonlinear data, and handling imprecise information [24]. The AI algorithm observed the data input from the subjective assessment (input layer), processed the information through the neurons to select the appropriate therapeutic exercises from the exercise bank (hidden layer), and then gave out the most appropriate therapeutic program (output layer). Figure 2 shows the architecture of the MLP-ANN of the AI used in this study. A total of 300 sets of training samples were initially provided by the experts during the expert meeting. Once the initial weighting of the MLP-ANN was trained by the samples provided by the experts, the back-propagation algorithm [25] was used to continue the training until an accuracy of at least 80% was achieved. Model validation was performed by comparing the AI-generated exercise program with the expert-generated exercise program.

The basic principle of the AI algorithm was based on the following: (1) each symptom was assigned a weighting in accordance to the clinical importance as determined by the expert panel (ie, the pain score had a higher weighting than social history); (2) each therapeutic exercise was assigned a weighting in accordance to their effectiveness to each of the presented symptoms as deemed by the expert panel; (3) the AI algorithm then selected the five highest scoring exercises from the database. Users were prompted to undertake reassessment every 14 days to report their progress and indicate the exercises that they found beneficial. The machine-learning algorithm would adjust the weighting of the exercise according to the individual’s feedback and update the exercise program based on the input of the reassessment. In the case of acute symptoms worsening, the algorithm would adjust the exercise plan according to the new symptoms. All AI-embedded mobile app users could contact members of the medical team who were involved in developing the app via the in-app messaging function. As part of the disclaimer of using the AI-embedded mobile app, users were advised that it did not replace medical service and medical support should be sought at any time should they feel the need to do so.

A points-based reward was utilized to promote engagement with the AI-embedded mobile app. Incentivized conditions have been shown to be effective in engaging users in goal-directed behavior [26]. Points were awarded according to the daily task completed (see Multimedia Appendix 2). The task list included logging onto the app, completing the number of sets of exercise, sharing their exercise progress on the social media account, and posting a comment about the educational material they read. Users needed to collect sufficient points to “unlock” the next level. The use of a point-based system aimed to increase engagement with the AI-embedded mobile app. The AI-embedded mobile app automatically recorded the number of days and the amount of time users spent on playing the exercise videos. An exercise log was kept and users could track their progress at any time.

#### Educational Component

Educational material was “pushed” to users via a social media platform once every 3 days. This material included information about neck and back pain pathology, the pathological cause of back pain and the natural course of low back pain, pain physiology, principles to use exercise as an intervention to manage neck and back pain, and coping strategies. These materials were stored within the app and users could access these materials at any time. Users received one reminder daily via the app reminder function which the user could turn off.

### Data Analysis

Data were analyzed in SPSS version 20.0 (IBM, Armonk, NY, USA) for Windows (Microsoft 10). Descriptive statistics were used to describe the sample population and gave a summary on all responses.

Changes in pain score and perceived self-improvement were analyzed as a group and as a subgroup based by the total duration of using the AI-embedded mobile app. Wilcoxon rank sum test was used to assess if the changes in Numerical Pain Rating Scale and self-perceived improvements were statistically significant.

## Results

### Overview

During the data collection period, the AI-embedded mobile app had 461 active users. A total of 161 users responded to the invitation. Three responses were excluded due to exclusion criteria (reported to be younger than 18 years of age). The sample population contained 119 males and 39 females. Of these, 30 and 31 responses reported to be in the age group of 18 to 25 years and 26 to 30 years, respectively. The age group between 31 and 40 years had the greatest number of responses (n=56). Nineteen and 21 responses reported to be in the age group of 41 to 50 years and 51 to 60 years, respectively. One response reported to be older than 60 years.

### Time Spent on Therapeutic Exercises

Most respondents reported they used the AI-embedded mobile app for a month. In all, 14.6% (23/158) and 13.3% (21/158) of respondents indicated they used the AI-embedded mobile app for 3 months and 6 months or more, respectively. The responses from question 3 of the evaluation questionnaire were 1 day (n=35), 1 week (n=38), 1 month (n=41), 3 months (n=23), and 6 months or more (n=21).

**Figure 3:**
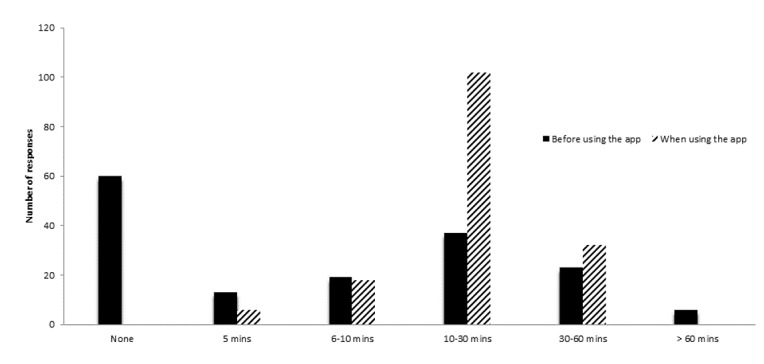
Bar graph to illustrate the self-reported time spent on therapeutic exercises before and when using the AI-embedded mobile app.

A total of 60 users (37.98%) reported they had never participated in therapeutic exercises prior to using the AI-embedded mobile app. When using the AI-embedded mobile app, the mean time spent on rehabilitation exercises was 25 (SD 4) minutes per day. An increased number of responses was observed in the categories of 10 to 30 minutes and 30 to 60 minutes, but there was a decreased number of responses for the more than 60 minutes category. Figure 3 is a bar graph showing the self-reported differences in time spent on therapeutic exercise before and when using the AI-embedded mobile app.

### Time Spent on Reading the Education Material

Overall, 142 users (89.9%) reported they had read the education material within the mobile app. Of these, 123 users (77.8%) indicated that the educational material had encouraged them to perform the AI-guided therapeutic exercise program. The mean time spent on reading the educational materials was 15 (SD 14) minutes a day.

### Numerical Pain Rating Scale

An overall reduction in the Numerical Pain Rating Scale score was reported from users after using the AI-embedded mobile app for therapeutic exercises. The median Numerical Pain Rating Scale scores were 6 (interquartile range [IQR] 5-8) before and 4 (IQR 3-6) after using the AI-embedded mobile app (95% CI 1.18-1.81). A Wilcoxon rank sum test indicated the reduction in Numerical Pain Rating Scale score was statistically significant (*P*=.04).

**Figure 4:**
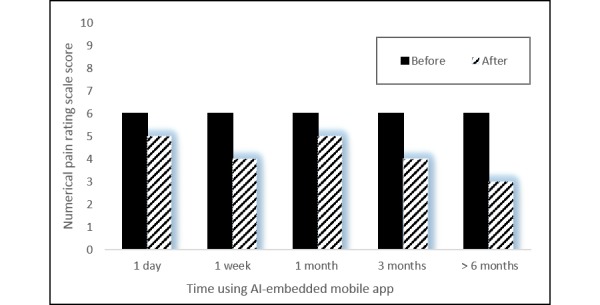
The reported difference in Numerical Pain Rating Scale changes reported by participants of different tenures.

The greatest reduction in Numerical Pain Rating Scale scores was reported from users who used the AI-embedded mobile app for 6 months. Figure 4 shows the changes in self-reported Numerical Pain Rating Scale scores reported by users with different tenures of using the AI-embedded mobile app.

### Self-Perceived Improvement

The mean self-perceived improvement from all users was 65%. Users who utilized the AI-embedded mobile app for 1 day, 1 week, and 1 month reported improvements of 60.2% (SD 27.9%), 49.5% (SD 29.2%), and 47.2% (SD 26.4%), respectively. For users who used the app for over 3 and 6 months, improvements were reported to be 58.6% (SD 30.4%) and 71.1% (SD 25.8%), respectively.

### Usage of Other Interventions

**Figure 5:**
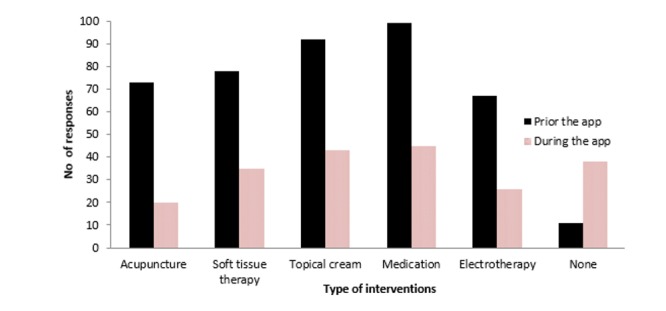
Bar graph to illustrate the number of responses of other interventions used prior to and when using the AI-embedded mobile app.

A total of 11 respondents (7.0%) indicated that they had not received prior intervention from the listed modalities and 38 respondents (24.1%) indicated they did not receive any other intervention while using the AI-embedded mobile app. A reduction in responses was observed in all the listed interventions when using the AI-embedded mobile app. Figure 5 shows the number of responses of who had used other interventions prior to and when using the AI-embedded mobile app.

### System Usability Scale

The median of the SUS from all responses was 73 (IQR 60-85), which would suggest an acceptable level of usability (score >68). All users, except for those who used the AI-embedded mobile app for a day (median 65, IQR 53-80), reported acceptable usability (score >68).

## Discussion

### Principal Findings

The main findings of this preliminary evaluation study were (1) users reported having spent more time on therapeutic exercises when using the AI-embedded mobile app, (2) an overall reduction in pain level was reported after using the AI-embedded mobile app, and (3) the usage of other interventions was reported to have been reduced with the use of the AI-embedded mobile app. This is among the first studies that investigated the feasibility and benefits of an AI-embedded mobile app for the management of chronic neck and low back pain.

### Adherence to Therapeutic Exercise

Exercise therapy had been recommended as a core component in the management of chronic neck and back pain by several guidelines and reviews [27-29]. It was an unexpected finding that 38% of the users indicated that they had never participated in therapeutic exercises before. It was possible that some of the users might live in a remote part of China where rehabilitation service was not widely available. The use of the AI-embedded mobile app may therefore play a role in increasing access to exercise therapy intervention, as supported by the reported increase in time engaged in therapeutic exercises. Adherence to a therapeutic exercise program in the management of chronic neck and low back pain has been a long-standing issue. Early literature showed that between 50% [30] and 70% [31] of people with chronic low back pain were nonadherent to a home exercise program. The use of technologies has been suggested to be a way to increase adherence to a home exercise program [32]. Previous studies that investigated the effectiveness of using mobile phone text message reminders indicated continued adherence to exercises in adults with recurrent low back pain [7]. The AI-embedded mobile app also had a reminder function that reminded users to perform daily exercise. A survey conducted in 2016 on the Chinese population with chronic low back pain (N=113) reported that self-management behaviors were poor [15] and contributing factors included lack of disease knowledge and lack of understanding of the benefit of exercise. The educational material may therefore play a role in promoting regular therapeutic exercise because it provides information on these areas. The majority of users indicated the material motivated them to do therapeutic exercise. The increase in self-reported time spent on exercises observed in this study may suggest its potential to be a useful tool for patient adherence to therapeutic exercise programs, which may contribute to improving clinical outcomes.

### Pain Level

A pain level reduction of 2 points (on a 0-10 Numerical Pain Rating Scale) is considered to be “much improved” [33] and a “meaningful” change [34] in people with chronic pain. The reduction in pain score was consistent with the finding of the Cochrane Review that indicated exercise, including motor control, flexibility, and strengthening exercises, may improve pain severity for people with chronic pain [35].

The difference in self-reported pain ratings was also consistent with a study that investigated the benefit of the combination of manual therapy and exercise therapy alone [36]. In this study, Alfuth and Welsink [36] investigated the pain level in people with chronic low back pain who attended physiotherapy practice for interventions. Interventions included a combination of manual therapy, stabilization exercises, and electrotherapy. A total of 85 participants received a mean number of treatment sessions of 42.6 (SD 2.3) within a year. A reduction of 3 points and 4 points on the Numerical Pain Rating Scale was observed after 3 and 6 months of treatment, respectively, when compared to baseline. Compared to the 2- and 3-point difference reported by users who used the AI-embedded mobile app for 3 and 6 months in this study, it would appear that using the AI-embedded mobile app to manage low back pain is feasible. An earlier study by Cambron et al [37] investigated the effect of a 4-week course of an active trunk extension exercise program at 3 months and 6 months postintervention. They reported a mean pain score reduction of 1.36 and 1.19 at 3 and 6 months postintervention, respectively. The difference in pain score reduction may be related to the type of exercises that participants were asked to do. The AI-embedded mobile app selected from a range of progressive exercises, whereas participants in the Cambron et al study [37] were only asked to do trunk extension exercises.

Users’ self-ratings of improvement of interventions is one of the core domains to be used in assessing impact of an intervention on chronic pain [38]. The self-rating of symptom improvement observed in this study corresponded to the reduction in pain score. The greatest symptom improvement was reported from users who had used the program for 6 months, followed by 3 months. A study published in 2010 reviewed 15 randomized controlled trials that were published between 2001 and 2007 [39]. The study summarized literature that reported long-term outcome data of physiotherapy exercise programs for adults with chronic low back pain. Trials were reviewed if they reported any outcome related to chronic low back pain measured at a follow-up period. Of the included trials, 13 reported follow-up data of up to 12 months and two trials reported follow-up data of up to 2 years. The results indicated consistent evidence for the effectiveness of an exercise program in pain reduction at the 6 months follow-up period when compared to a control group. Our study also observed the greatest self-reported pain score reduction and self-perceived improvement from users who used the AI-embedded mobile app for 6 months.

When compared to the only other trialed mobile app (FitBack) [40] that focused specifically on spinal pain management [10], the AI-embedded mobile app used in our study appeared to be at least as effective in reducing pain level. FitBack users reported a reduction in pain score from 2.86 at baseline to 2.64 and 2.16 at follow-up at 2 and 4 months, respectively. A Cochrane Review [35] and the NICE guideline [21] indicated that exercise programs should be tailored according to individual needs and capabilities. Therefore, the higher reduction of pain scores observed in this study may be related to the tailoring of the exercise content based on the presenting symptoms. But the higher magnitude of pain score reduction observed in our study should be interpreted with caution. The two studies have different designs, which may contribute to the differences in reported pain levels. It is typical for single-group data to show greater effects than randomized controlled trial data. The pain score data from our study may also contain recall bias [39], which further complicates the comparison.

### Needs for Other Interventions

The results of our study indicate that using the app for the self-management of chronic neck and back pain might reduce the need for other interventions. The reported reduction in usage of other interventions was supported by an earlier study by Sculco et al [41]. Their study investigated the effect of 10 weeks of a prescribed home-based exercise program in people with chronic low back pain compared to a control group. The results suggested that participants in the exercise group had significantly lower numbers of physiotherapy referrals and medication use during the 2.5-year follow-up period. The reported reduction of other intervention usage suggests that the AI-embedded mobile app may at least reduce the ambulatory time to medical appointments, thus reducing the indirect health care costs that are associated with seeking intervention. Further study is recommended to understand its cost benefit within the local health care system.

### Limitations

The results reported here must be viewed cautiously due to the limitations. This was a single-group study with no control which relies heavily on self-reported subjective data. This is among the first AI-embedded mobile apps developed for the management of low back pain. Thus, the primary goal of this preliminary study was to evaluate the perceived benefits of such a system by its users. With regard to the fidelity of the intervention, it is worth considering that the app was available for download by members of the general public. Users were not advised by anyone to download it; they downloaded it on their own initiative, assuming that they suffered from chronic neck and back pain and were looking for self-help material on the Web. Thus, it is reasonable to believe that users had followed the therapeutic program provided by the AI-embedded mobile app. This study defined the term “adherence” as the amount of self-reported time spent on the exercise generated by the AI-embedded mobile app. Therefore, the interpretation of adherence within this study cannot be generalized to the long-term adherence to therapeutic exercise. The “law of attrition” of eHealth trials [42] did not appear to be applicable in this study because this study only recorded response rate but not dropout rate. We could not verify whether participants’ eligibility criteria information were accurate, which may affect their responses to the intervention. The amount of time participants viewed the video clips were not verified. However, the AI-embedded mobile app has a scoring system which the user had to satisfy by playing the video clip for the exercises. Further study that combines this with medical verification would provide greater confidence in the intervention effects. This study did not evaluate the functional aspect of the intervention. Thus, it was unclear if the changes in pain score may translate to improvement in function. We included active users at the time of the study, which many have affected the response rate. The study design also had sample selection bias because it relied on people who responded to the invitation, thus the sample population would be more likely to participate in the intervention. This was a retrospective evaluation study, which was likely to be associated with recall bias of preintervention symptoms and behaviors. The focus of this preliminary study was to assess the self-perceived benefits from users. Self-reported data on health care utilization are commonly used in health care research [43]. Self-reported data may be associated with recall bias; however, a study that investigated agreement between self-reported health care service usage and administrative records indicated good agreement between the two [44].

### Conclusions

The key findings of this evaluation study support the perceived beneficiary effects of the AI-embedded mobile app to provide some personalized interventions that are tailored to individual needs for the self-management of chronic neck and back pain. The positive results of this study suggest that using the tool to assist the self-management of chronic spinal pain may be feasible. The tool at least warrants further study to investigate the benefit of the AI-embedded mobile app and how it may compare with routine clinical care. Further study is also recommended to understand if the AI-embedded mobile app may induce long-term sustainable behavioral change.

## Appendix

Multimedia Appendix 1 Screenshots of a detailed description of the exercise (left) and of the annotated diagrams to show the targeted muscles (right).

Multimedia Appendix 2 Screenshots of the daily task list (top) and the exercise log where users could track their progress (bottom).

## Abbreviations

AI: artificial intelligence
eHealth: electronic health
ICT: information and communication technology
IQR: interquartile range
MLP-ANN: multilayered perceptron artificial neural network
NICE: National Institute of Clinical Excellence
NPRS: Numerical Pain Rating Scale
SUS: System Usability Scale
