# Supplementary figures and images for: The Perceived Benefits of an Artificial Intelligence–Embedded Mobile App Implementing Evidence-Based Guidelines for the Self-Management of Chronic Neck and Back Pain: Observational Study
Source: JMIR Mhealth Uhealth. 2018 Nov 26;6(11):e198. doi: 10.2196/mhealth.8127 (PMC6288595; doi:10.2196/mhealth.8127)

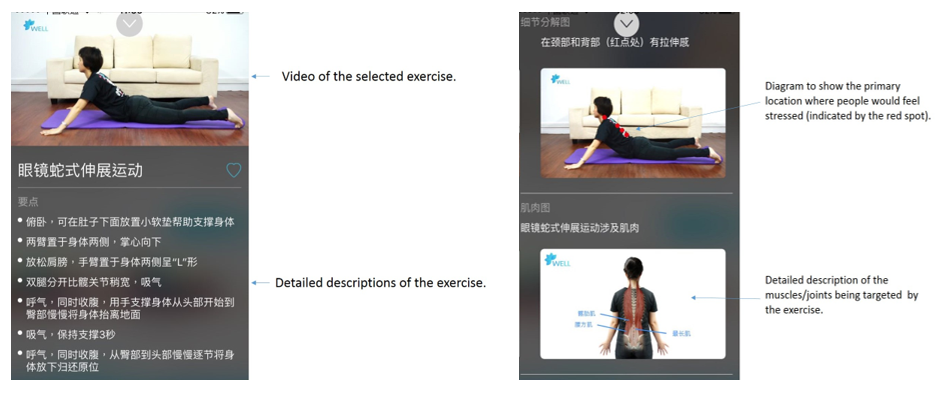

Supplement: Multimedia Appendix 1 [file mhealth_v6i11e198_app1.png]

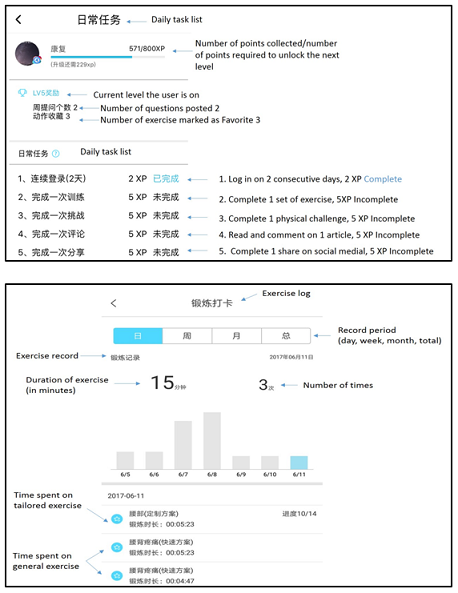

Supplement: Multimedia Appendix 2 [file mhealth_v6i11e198_app2.png]
